# Inertial Motion Capture-Based Whole-Body Inverse Dynamics

**DOI:** 10.3390/s21217353

**Published:** 2021-11-05

**Authors:** Mohsen M. Diraneyya, JuHyeong Ryu, Eihab Abdel-Rahman, Carl T. Haas

**Affiliations:** 1 Institute for Aerospace Studies, University of Toronto, 4925 Dufferin Street, North York, Toronto, ON M3H 5T6, Canada; m.diraneyya@mail.utoronto.ca; 2 Department of Civil and Environmental Engineering, University of Waterloo, 200 University Avenue West, Waterloo, ON N2L 3G1, Canada; chaas@uwaterloo.ca; 3 Department of System Design Engineering, University of Waterloo, 200 University Avenue West, Waterloo, ON N2L 3G1, Canada

**Keywords:** inertial motion capture (IMC), inverse dynamics, joint load, ergonomics, physical exposure

## Abstract

Inertial Motion Capture (IMC) systems enable in situ studies of human motion free of the severe constraints imposed by Optical Motion Capture systems. Inverse dynamics can use those motions to estimate forces and moments developing within muscles and joints. We developed an inverse dynamic whole-body model that eliminates the usage of force plates (FPs) and uses motion patterns captured by an IMC system to predict the net forces and moments in 14 major joints. We validated the model by comparing its estimates of Ground Reaction Forces (GRFs) to the ground truth obtained from FPs and comparing predictions of the static model’s net joint moments to those predicted by 3D Static Strength Prediction Program (3DSSPP). The relative root-mean-square error (rRMSE) in the predicted GRF was 6% and the intraclass correlation of the peak values was 0.95, where both values were averaged over the subject population. The rRMSE of the differences between our model’s and 3DSSPP predictions of net L5/S1 and right and left shoulder joints moments were 9.5%, 3.3%, and 5.2%, respectively. We also compared the static and dynamic versions of the model and found that failing to account for body motions can underestimate net joint moments by 90% to 560% of the static estimates.

## 1. Introduction

Inverse dynamic analysis is used in biomechanics to predict the forces and moments developing in muscles and joints during body motions. It requires experimental measurements of a range of data, namely the subject anthropometric data, their motion kinematics, and the external forces applied to the body during motion [1,2].

Optical motion capture (OMC) systems have historically been considered the gold standard for kinematic measurements. However, optical tracking requires a line-of-sight between the cameras and body markers [3,4] or body segments, which complicates the experimental set-up and constrains their use to confined laboratories [2]. These constraints render OMC systems impractical for on-site studies and other tasks that are performed in open space.

Inertial motion capture (IMC) systems—a composite of inertial measurement units (IMUs)—are an alternative solution. IMC systems continuously capture whole-body motions using measured acceleration, angular rate, and magnetic field orientation [5,6]. They are portable and eliminate the need to maintain line-of-sight between the camera and markers or body segments. The fidelity of motion kinematics obtained from IMC systems has been previously verified [7,8,9,10,11,12,13]. Currently available IMC systems have been used in different domains for various applications, including activity recognition [14,15,16] and activity assessments [17,18,19]. Marta et al. [17] developed a wearable biofeedback suit equipped with 10 IMUs. Using the suit and a custom-made algorithm, they analyzed real-time joint angles to evaluate performance during aquatic exercise.

Those efforts showed potential to use IMC systems for kinematic analysis. However, only a few studies have used motion kinematics in kinetic analysis to predict joint loads or muscle forces [1]. For example, Kim and Nussbaum [3] compared the shoulder, knees, hips, and L5/S1 joint angles and moments calculated by inverse dynamics models based on kinematic data obtained from an OMC system to those based on kinematic data obtained from an IMC system. The mean absolute error between the two sets of results across various manual material handling tasks was less than 16.4 N·m for joint moments. One limitation to this approach is its dependence on force plates (FPs) for ground reaction force (GRF) measurements, which undermines the portability of IMC systems.

Typically, the external forces acting on the body consist of GRFs and hand loads. While hand loads can be easily estimated based on the task at hand or measured via instrumented gloves [20], GRF measurements are challenging to collect. The standard method to measure them, FPs, is unsuitable for out-of-lab experiments in unstructured environments because of their needs for ground mounting and limited capture area [21]. To remove this barrier, methods have been proposed to estimate GRFs using kinematic data measured from IMC systems. Faber et al. [1] estimated GRFs using a top-down inverse dynamics approach based on motions obtained from IMC system. They compared their estimate of the GRFs with FP measurements and found that their results were in close agreement for the dominant components of the GRFs (vertical) with coefficients of determination value above 0.981. However, the model did not break down the total GRFs into right and left foot contributions; therefore, it cannot be used to study the lower extremities’ kinetics.

The great majority of algorithms estimating GRFs from kinematic data are limited to the single-foot support phase during walking and running [22,23]. In that phase, the GRF of the foot contacting the ground is also the total GRF. The problem becomes statically indeterminate during the double-stance phase, where both feet are in contact with the ground, and is yet to be addressed effectively [24,25]. Several studies have attempted to solve it by estimating the breakdown into right and left GRFs using empirical functions [26,27,28], machine learning [29,30], or optimization [31]. However, most of these studies use FP measurements to detect the start and end of the double support phase, which undermines the value of their approaches as alternatives to FPs. Karatsidis et al. [28] proposed a gait event detection algorithm to overcome this shortcoming. It predicts foot contact with the ground by comparing heel and toe velocities to the pelvis velocity. They divided the GRF during gait into right and left GRFs using a smooth transition assumption. Over normal walking speed, the IMC predicted and FP measured GRFs were in good agreement with relative root-mean-squared errors (rRMSE) of 5.3%, 9.4%, and 12.4% for the vertical, anterior-posterior, and medial-lateral components of GRFs, respectively. However, their algorithm was limited to gait only and was unable to handle other tasks that included aperiodic movement patterns or external loads.

This study aims to develop a whole-body inverse dynamics model that is applicable to arbitrary motion patterns captured by an IMC system without a need for FPs or other specialized equipment. Particularly, we develop and demonstrate a contact detection algorithm to predict foot contact and an optimization approach to decompose total GRFs into right and left GRFs during the double-stance phase. The present study contributes to the body of knowledge on estimation of kinetic parameters based on kinematics obtained from an IMC system only. Additionally, the findings of this study enable the use of inverse dynamics to analyze a variety of tasks in any setting, including outdoors.

## 2. Inverse Dynamics Model

**Figure 1:**
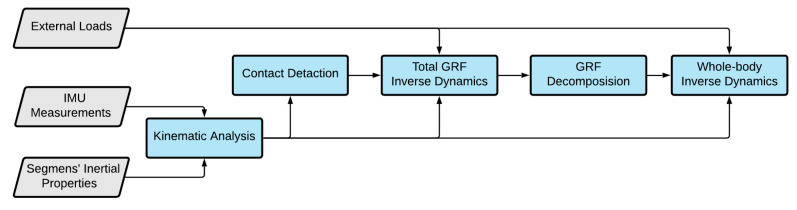
Flowchart of the whole-body inverse dynamics model.

The overall structure of the whole-body inverse dynamics is shown in Figure 1, in which input to the model appears in grey parallelograms and blue rectangles represent the processing steps carried out in MATLAB. The input IMU measurements are (1) Biovision Hierarchy (BVH) files containing the location of the pelvic joint center in the reference frame and the rotation matrices between each segment frame and its parent frame and (2) Calculation (CALC) files containing the locations, accelerations, and angular velocities of the IMUs in the reference frame.

**Figure 2:**
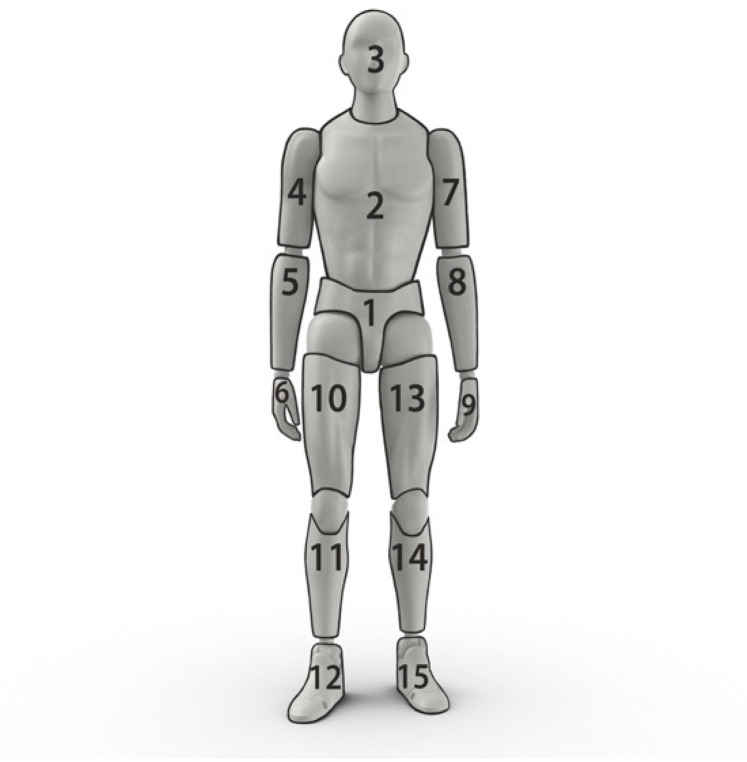
The segments of the whole-body model.

We model the human body as a rigid-body system composed of 15 segments: the pelvis, torso, head, right and left upper arms, forearms, hands, thighs, shanks, and feet, see Figure 2. Following the International Society of Biomechanics (ISB) standard coordinate system [32,33], we used the subject’s height and weight to obtain the segments’ inertial properties, namely length and mass, location of the center of mass, and the second moment of mass, from the expressions derived by Dumas et al. [34]. The external forces and moments applied to the body are either active or reactive. Active forces and moments are evaluated as per the activity under study, e.g., hand loads during manual handling. The only reactive forces and moments are GRFs and Ground Reaction Moments (GRMs) while standing or walking.

The internal forces and moments in the model are the net joint forces and moments, segment weight, and inertial forces. To evaluate the latter, the acceleration of segment *i*’s center of mass $a^{i}\left(t\right)$ was calculated as:

(1) $$a^{i}\left(t\right)=a_{s}^{i}-\alpha^{i}\times r_{s}^{i}-\omega^{i}\times \left(\omega^{i}\times r_{s}^{i}\right).$$

where $a_{s}^{i}$ is the acceleration measured by the segment fixed IMU, $r_{s}^{i}$ is the location of the IMU with respect to the center of mass, and $\omega^{i}$ and $\alpha^{i}$ are the segment angular velocity and acceleration, respectively.

Two inverse dynamics problems are solved starting from the distal segments (hands and feet) and propagating inward to the pelvis, by solving for the proximal net joint force and moment of each segment using its equations of motion. In other words, a top-down approach is taken for upper-body segments and a bottom-up approach is taken for the lower-body segments. The top-down problem starts from the measured weight of hand-held objects, whereas the bottom-up problem starts from an estimate of the GRFs.

### 2.1. Total Ground Reaction Force

Assuming no other reaction forces, the total GRF $F_{g}$ can be calculated as the sum of all body segments’ inertial forces $F^{i*}$, the weights of all body segments $W^{i}$, and external forces $F_{ex}^{i}$:

(2) $$F_{g}=\overset{15}{\underset{i=1}{\sum }}\left(F^{i*}-W^{i}-F_{ex}^{i}\right)$$

Similarly, the total GRM can be calculated as the sum of all inertial moments $M^{i*}$, the moment produced by all inertial forces, the moment produced by all body segment weights, external moments $M_{ex}^{i}$, and the moment produced by all external forces:

(3) $$M_{g}=\overset{15}{\underset{i=1}{\sum }}\left(M^{i*}+l^{i}\times F^{i*}-l^{i}\times W^{i}-M_{ex}^{i}-l_{ex}^{i}\times F_{ex}^{i}\right)$$

where $l^{i}$ is the location of segment i center of mass with respect to the pelvic frame origin. This formulation distinguishes $F_{g}$ and $M_{g}$ from the external forces and moments, respectively, acting on the body.

When one foot only is in contact with the ground, this problem is deterministic and $F_{g}$ is equal to the foot contact force. However, during the double-stance phase, a method is required to divide the $F_{g}$ into right and left GRFs. Therefore, it is necessary to determine first whether both feet are in contact with the ground. Where that is found to be the case, we need to solve an indeterministic problem to distribute $F_{g}\;$ into right and left GRFs.

#### 2.1.1. Contact Detection

**Figure 3:**
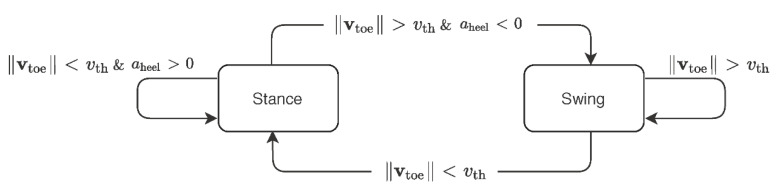
Ground contact detection algorithm.

Following Karatsidis et al. [28], ground contact was predicted by comparing toe and heel speed as well as the magnitude of their velocities, at each time step, to a threshold speed of $v_{th}$. While Karatsidis et al. [28] defined $v_{th}$ as the magnitude of the average velocity of the pelvis segment for each trial, we set $v_{th}$ to 1.2 m/s, which is the average walking speed of a healthy adult [35]. The foot is assumed to be in contact with the ground, if the toe speed drops below the threshold $||v_{toe}||<v_{th}$. Conversely, the foot loses contact with the ground when the toe speed exceeds the threshold $||v_{toe}||\geq v_{th}$. The threshold speed is taken as the average forward (pelvis) speed over the experiment to account for noise in the velocity measurements. Figure 3 shows a state diagram of the foot contact algorithm.

Unlike Karatsidis et al. [28], we simplified the conditions on stance and swing phases by using the common threshold speed and imposing it consistently on the toe speed. This allows us to better incorporate the toe-off stage within the stance phase. Furthermore, to distinguish between the toe-off and leg-lift stages, we introduced a condition that terminates the stance phase when the heel acceleration $a_{heel}$ switches sign from positive to negative. The heel acceleration $a_{heel}$ was obtained here by differentiating the heel speed, and thus, it indicates switching from increasing to decreasing speed. This condition enforces the assumption that the heel reaches maximum velocity at toe-off.

#### 2.1.2. Decomposition of the Ground Reaction Force

We overcame the indeterminacy during double-stance phase by solving an optimization problem [31] to estimate the force breakdown between right and left GRFs. The moment-based cost function seeks to minimize the sums of the squared of the net joint moments, minimizing the energetic cost, in the closed kinematic stance loop, namely the right and left ankles $M_{ra}$ and $M_{la}$, right and left knees $M_{rk}$ and $M_{lk}$, and right and left hips $M_{rh}$ and $M_{lh}$:

(4) $$\min\left\{{||M_{ra}||}^{2}+{||M_{la}||}^{2}+{||M_{rk}||}^{2}+{||M_{lk}||}^{2}+{||M_{rh}||}^{2}+{||M_{lh}||}^{2}\right\}$$

The underlying assumption of the cost function is that the neuromuscular system acts to minimize the muscle effort, represented by the net joint moments, required to maintain the stance [36,37,38]. The net moments were minimized and subject to:

- Two equality constraints that ensure the equations of motion are satisfied by equating the sum of the right and left GRFs and GRMs to the total GRF and total GRM, obtained from the equations of motion (5) and (6):
  (5) $$F_{rg}+F_{lg}=F_{g}$$
  (6) $$M_{rg}+M_{lg}=M_{g}$$
  where $F_{rg}$ and $F_{lg}$ are the right and left ground reaction forces and $M_{lg}$ and $M_{lg}$ are the right and left ground reaction moments, respectively.
- One inequality constraint to ensure that the vertical components of GRFs are always pointing upward:
  (7) $$F_{rg}>0\;\&\;F_{lg}>0$$

## 3. Research Methods

### 3.1. Experiment

**Figure 4:**
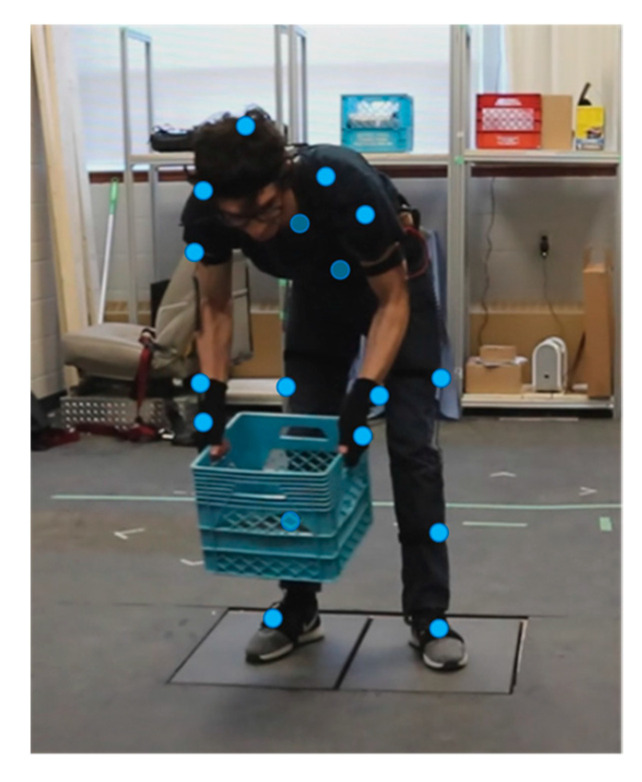
Photo of a subject wearing the Perception Neuron [39] IMC system while performing a lifting task; IMU locations are marked with blue dots.

Three healthy subjects participated in the experiment consisting of two adult males and one adult female. The mean and standard deviation of their age, height, body mass, and body mass index are 26.7 (±4.7) years, 177.3 (±6.4) cm, 78.9 (±16.6) kg, and 25 (±4.34), respectively. Each participant performed three trials of three tasks:

(1) Walking: Participants performed one gait cycle such that each foot landed on the corresponding FP.
(2) Jumping: Participants jumped onto the FP from a stationary standing posture approximately 0.25 m behind the FPs.
(3) Lifting (manual material handling): The experiment comprised three full handling cycles. At the beginning of each cycle the participant stood stationary with each foot on the corresponding FP. Then, the participant stooped while leaning to the left to pick up a box weighing 17 kg (dimensions 0.34 × 0.34 × 0.27 m), proceeded to move it, see Figure 4, to a corresponding location on the right side, and deposited it there, before returning to their initial position of stationary stance. To complete the cycle, the participant repeated the same steps to move the box back from front right to its home station at the front left. Figure 4 also shows a subject wearing the IMC system and highlights the IMU locations with blue dots.

The study protocols were approved by the Research Ethics Committee at the University of Waterloo.

### 3.2. Instrumentation and Data Pre-Processing

We utilized the Perception Neuron [39] IMC system to acquire full-body motion data. The system consists of 17 IMUs, each unit being composed of a three-axis accelerometer, a three-axis gyroscope, and a three-axis magnetometer. The measurement ranges of the accelerometer and gyroscope are ±16 g and ±2000 °/s, respectively. The accuracy of the joint kinematics estimates obtained from this IMC systems was found comparable to that of optical motion capture systems [40]. Specifically, they found that the IMC system had an acceptable level of error, under 5°, particularly in the sagittal plane.

The IMUs were firmly attached to the participant’s head, back, shoulders, upper arms, forearms, hands, thighs, legs, and feet using elastic straps. We recorded body motions at a sampling rate of 120 Hz via the companion motion capture software, Axis Neuron^14^. At the beginning of the experiment, participants carried out a calibration step to determine the sensor-to-body alignment and body dimensions directed by the motion capture software, which required each participant to take a T-pose, an A-pose, and an S-pose.

The right and left GRFs were measured using two force plates (FP4060-05-PT-1000, Bertec Corporation, Columbus, ON, USA) at a sampling rate of 960 Hz. To synchronize the measurements from the FPs and the IMC system, an OMC system (Vantage plus, Vicon, Oxford, UK) was employed to track the position of a single marker attached to the pelvic anterior-superior iliac spine marker throughout the experiment. The FPs and OMC data were measured synchronously on one computer and the IMC data were synchronized by detecting distinguished features in a stationary pose and in the motion of the pelvic anterior-superior iliac as recorded by both OMC and IMC.

The measured full-body motions and GRFs were filtered using a second-order zero-phase Butterworth low-pass filter with a cut-off frequency of 10 Hz. The GRFs were then sampled down to 120 Hz to match the sampling frequency of the kinematic data.

### 3.3. Statistical Analysis

We examined the efficacy of the proposed method by comparing the predicted left and right GRFs to those measured by the FPs. We also validated our model by comparing its estimates of the static net joint moments to those obtained from an established software for static biomechanical analysis, the 3D Static Strength Prediction Program (3DSSPP) [41]. The latter calculates compression and shear forces in the lumbar joint at the L4/L5 disc level and the net joint moments in the elbow, shoulder, L5/S1 disc, hip, and knee using a top-down approach that starts from the forces and moments applied to the hands and ends with the forces and moments applied to the floor by the feet. To run this analysis, we converted the captured motion files to joint location files describing the whole-body joint center locations in a global coordinate system (X, Y, and Z coordinates) using an in-house MATLAB code [19,42].

The root-mean-squared error (RMSE) and rRMSE were used as metrics to evaluate the agreement between the two sets of results over given tasks, namely predicted GRFs versus measured GRFs and static joint loads predicted by our model versus those predicted by 3DSSPP. The RMSE is defined as:

(8) $$RMSE=\sqrt{\frac{1}{T}\int_{0}^{T}(x_{1}\left(t\right)-x_{2}{\left(t\right)}^{2})dt}$$

where $x_{1}$ is a measurement, $x_{2}$ is the corresponding model prediction, and *T* is the total task time. The rRMSE is also defined as:

(9) $$rRMSE=\frac{RMSE}{max\;\left(\left|x_{1}\left(t\right)\right|\right)}\times 100\%$$

Furthermore, for each task, we compared the peak values extracted from the time series of the reference system (FPs or 3DSSPP) to the peaks obtained from our model for all trials and subjects using intraclass correlation coefficients (ICCs) [43]. The ICCs were categorized as poor (ICC ≤ 0.5), moderate (0.5 ≤ ICC ≤0.75), good (0.75 ≤ ICC ≤ 0.9), and excellent (0.9 < ICC) [44].

## 4. Results

### 4.1. Ground Reaction Forces

**Figure 5:**
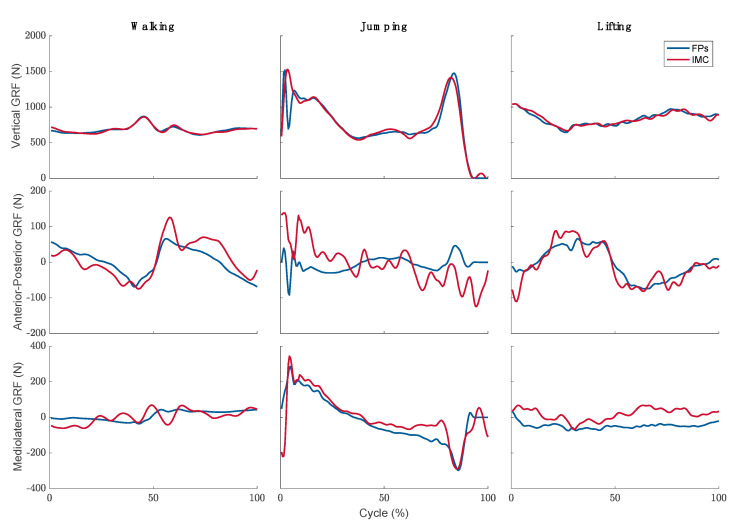
Typical examples of the FP measured (blue line) and IMC predicted (red line) vertical, anterior-posterior, and mediolateral components of the total GRF during walking, jumping, and lifting.

Typical examples of the vertical, anterior-posterior, and mediolateral components of the total GRF measured by the FPs and predicted by the dynamic model during walking, jumping, and lifting are shown in Figure 5. The measured total GRF time-series is shown in blue, whereas the predicted total GRF is shown in red.

Estimates of the vertical component of the total GRF are in excellent agreement with the measurements for all three tasks as indicated by their ICC. Taking the measured GRF as the ground truth, we calculated the RMSEs and rRMSEs of the predicted GRFs as listed in Table 1. We found that the rRMSEs were consistently small at 3.57% for walking, 6.08% for jumping, and 8.31% for lifting, in agreement with ICC results. However, estimates of the other components revealed large rRMSEs, ranging from 51.68% to 182.59% for the anterior-posterior component and from 26.62% to 141.31% for mediolateral component.

**Figure 6:**
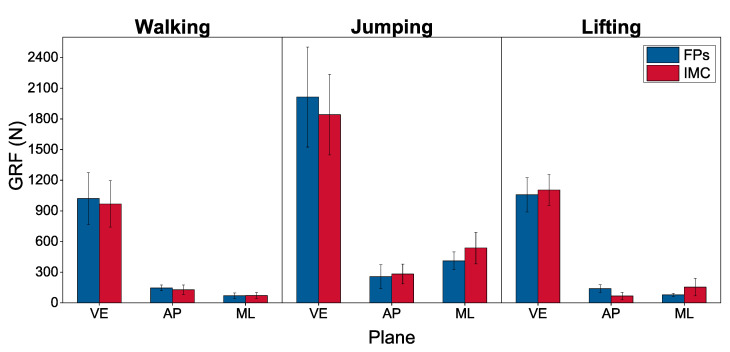
Average peak FP measured (blue bars) and IMC predicted (red bars) components of the total GRF during walking, jumping, and lifting; VE = vertical component, AP = anterior-posterior component, ME = mediolateral component.

The magnitudes of the predicted and measured peaks of the total GRF components are shown in Figure 6 averaged over all trials by subjects. The vertical component of the total GRF was dominant across all three tasks. There was also excellent agreement between the measured and estimated values of this component. The other two components were much smaller than the vertical component with their combined magnitude reaching a maximum 24% (measured) and 33% (predicted) of the vertical component for jumping.

**Figure 7:**
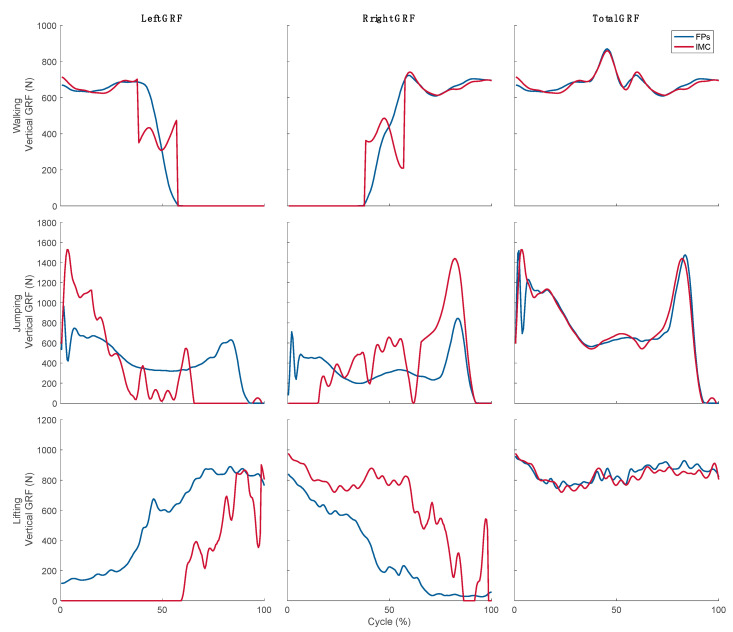
Typical examples of the FP measured (blue line) and IMC predicted (red line) vertical components of the left, right, and total GRFs during walking, jumping, and lifting.

Figure 7 shows the FP measured and IMC predicted vertical component of the total GRF and its breakdowns into right and left GRFs during walking, jumping, and lifting. Model predictions of the left and right GRFs were in good agreement with FP measurements during the single-foot stance phases of walking. The peak around mid-cycle corresponds to heal strike, as the impact increases the vertical component of the total GRF, and demarcates the start of the double-stance phase. Model predictions of the GRF breakdown during that phase were less accurate. On the other hand, both feet were in contact with the ground throughout the lifting and jumping tasks. In those cases, the model interprets weight shifting from one foot to the other as non-contact on the non-weight bearing foot and predicts this process successfully.

Quantitative comparison of the predicted and measured right and left GRFs reveal similar patterns to those of the total GRF. The predicted and measured vertical components of the left and right GRFs during walking were in good agreement as per their ICCs, with rRMSE values at 16.61% for the right foot and 16.37% for the left foot. Agreement between the measured and predicted left and right vertical components of the GRFs was poor during jumping and moderate during lifting as per their ICCs. The corresponding rRMSE values were 38.8% for the right vertical GRF and 48.51% for the left vertical GRF during jumping and 46% to 47% for the right and left vertical GRFs during lifting. Predictions of the anterior-posterior and mediolateral components were poor for all three tasks.

### 4.2. Model Validation

**Figure 8:**
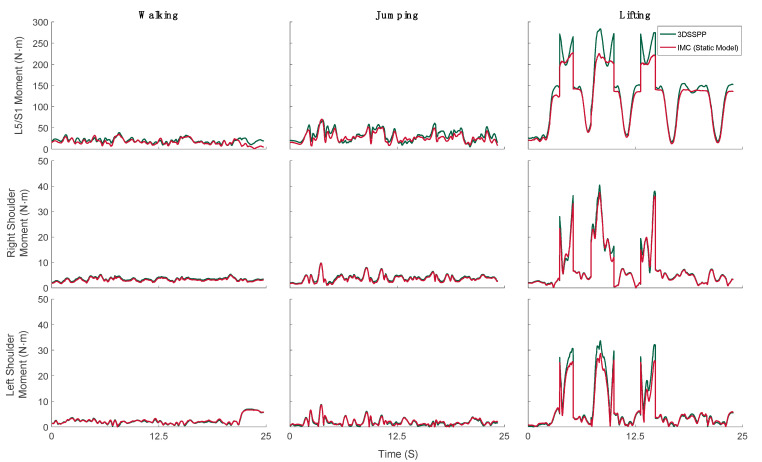
Typical examples of 3DSSPP-calculated (green lines) and IMC model-predicted (red lines) net static moments of L5/S1, right, and left shoulder joints during walking, jumping, and lifting.

We compared the net moment of the L5/S1, right shoulder and left shoulder joints, which are the joints primarily affected by the tasks under study. Typical examples of the net moment of the L5/S1 and the right and left shoulder joints during walking, jumping, and lifting are shown in Figure 8, where joint moments calculated by 3DSSPP are shown as green lines and those predicted by our IMC model are shown as red lines. 3DSSPP evaluates only the net reaction moment resulting from applying the static loads of the segment weights and external static forces, the box weight during the lifting task, to the current body posture. It does not, however, consider inertial forces due to accelerations. To mimic these conditions, a static IMC model was derived by setting the accelerations of body segments equal to zero. This model was then used to estimate the static net moment at the L5/S1 and the right and left shoulder joints.

Both sets of estimates of the net joint moments were in excellent qualitative agreement and good quantitative agreement for all three tasks. Taking the 3DSSPP-calculated net joint moments as the ground truth, we calculated the RMSE, rRMSE, and ICC values of those predicted by the IMC as listed in Table 2. The maximum rRMSE was 11.85% during walking at the L5/S1.

**Figure 9:**
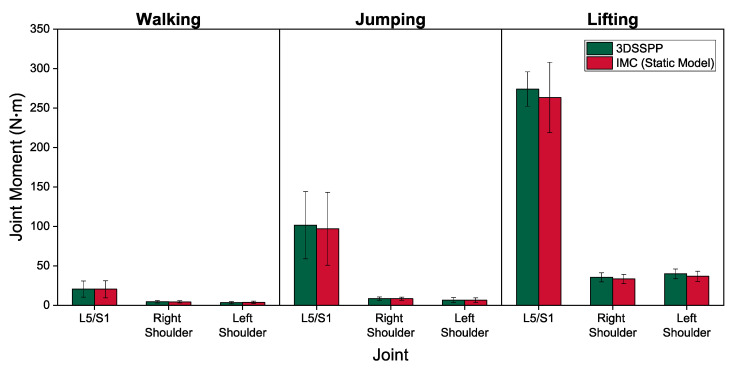
Average peak 3DSSPP-calculated (green bars) and IMC model-predicted (red bars) net static moments of L5/S1, right, and left shoulder joints during walking, jumping, and lifting.

The magnitudes of the peak joint loads estimated from 3DSSPP and our model, shown in Figure 9, were averaged over all trials by all subjects. The net static joint moments showed excellent correlations for all tasks, with the ICC ranging from 0.93 to 0.99, except for the L5/S1 joint, where the agreement was good, with ICC = 0.90.

### 4.3. Dynamic Biomechanical Analysis

**Figure 10:**
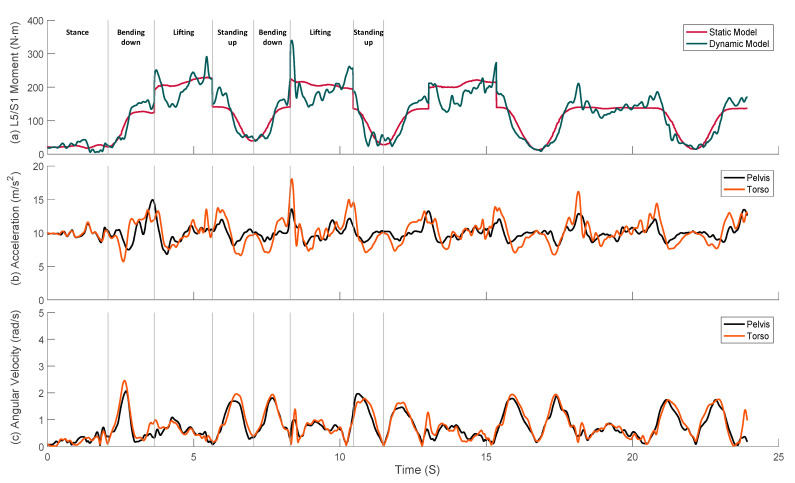
Typical examples of (**a**) predicted net L5/S1 joint moments by static (red line) and dynamic (dark-green line) models, (**b**) corresponding center of mass acceleration, and (**c**) angular velocities of the pelvis (black lines) and torso (orange lines) during lifting.

**Figure 11:**
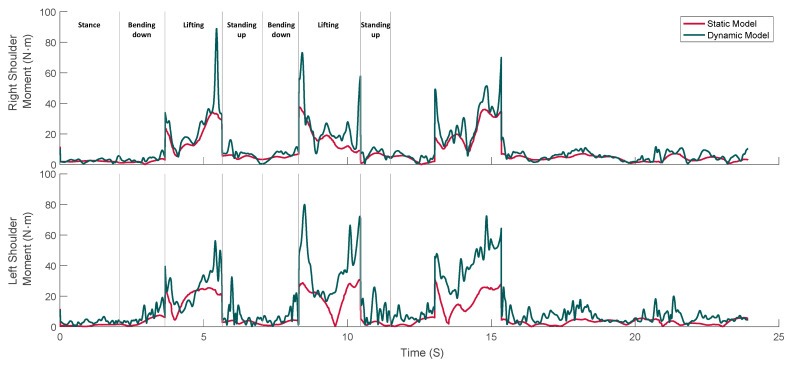
Typical examples of predicted right and left shoulder joint moments by the static (red lines) and dynamic (dark-green lines) models during lifting.

To investigate the additional loads imposed by participant motions (inertial loads), we compared the estimates of joint loads obtained from the static model described above those obtained from the dynamic model. Figure 10a and Figure 11 show the predictions of the net L5/S1 joint moment and the right and left shoulder joint moments during lifting, respectively. The static model estimates are shown in red lines and the dynamic model estimates are shown in dark-green lines. Furthermore, based on the recorded video, we manually divided the lifting task into bending down, lifting, and standing up phases. The ‘bending down’ phase starts when the subject initiates torso flexion and ends when the subject wraps their hands around the box handles. The ‘lifting’ phase starts when the subject applies upward forces to the box until they place it entirely on the ground at the other side. Lastly, the ‘standing up’ phase starts as the subject releases their grip on the box handles and ends when they come to rest fully erect.

Predictions of both models were in qualitative agreement, but those of the dynamic model showed significant excursions away from a mean trend prescribed by the static model for all three joints. Examination of the time-histories of segment motions shows that those excursions are closely related to accelerations of related body segments. Figure 10b,c show the corresponding center of mass acceleration and angular velocity of the pelvis (black lines) and torso (orange lines). Excursions in the dynamic estimate of the L5/S1 joint moment away from the static estimate are synchronous with excursion in the torso’s center of mass acceleration compared to that of the pelvis. Specifically, the higher relative (to pelvis) torso acceleration at the start and end of the lifting stage caused higher net L5/S1 joint moment, while the lower acceleration levels experience at the mid-lifting stage produced lower net joint moment compared to the static loads. On the other hand, no obvious correlation was observed between the variation of the segment angular velocity and the net joint moments.

Significant excursions in the dynamic estimates to the static estimates of net joint moment were also found at the shoulder joints, see Figure 11. In particular, the arms accelerate with respect to the torso at the beginning and end of the lifting stage; thereby, the dynamic to static load ratio increases at these instances. Furthermore, an instantaneous impact event occurs when depositing the 17-kg box on the ground at the end of the lifting stage. This event was observable in the dynamic estimates of the net shoulder joint moments, as one or more sharp peaks occur towards the end of the lifting stage, but not in the corresponding static estimates.

**Figure 12:**
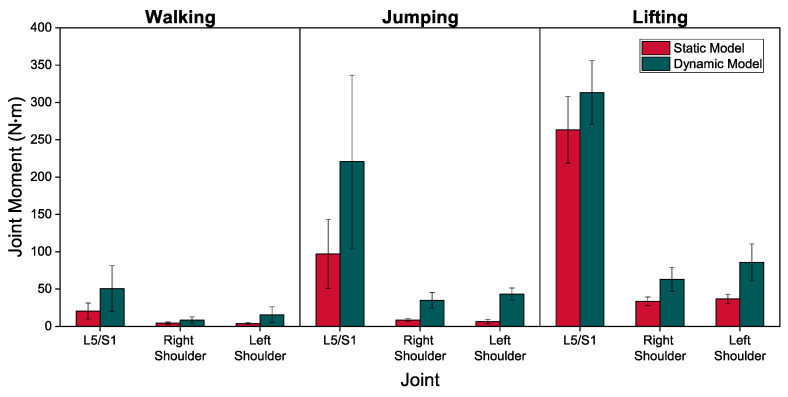
Average peak of the net moments of L5/S1, right, and left shoulder joints predicted by static (red bars) and dynamic (dark-green bars) models during walking, jumping, and lifting.

Figure 12 shows the average peak of the net static and dynamic joint moments during walking, jumping, and lifting. The dynamic moment peaks are larger than the static peaks for all activity and joints, with the ratio ranging from 1.19 to 6.61. This is expected since the dynamic estimates account for inertial loads not captured in the static model. Furthermore, it is noted that the dynamic estimates of joint moments are larger than the static estimates for jumping and lifting compared to walking. This is expected, since jumping involves higher segment accelerations than walking and impact with the ground and lifting involves moving a significant mass, in addition to body segments, as opposed to the previous two cases.

## 5. Discussion

Estimating the biomechanical loads on the human body is a tedious process that requires a laboratory environment, stationary equipment (force plates and motion capture cameras), and a dedicated space to capture body motions and GRFs. However, the use of IMC systems can reduce these requirements significantly and allow for biomechanical task evaluation outside of the lab environment.

This study developed a whole-body inverse dynamic model that estimates the net moments in major body joints using an IMC system only. To overcome the indeterminate problem of estimating GRFs during double-stance phase, we developed a contact detection algorithm to predict foot contact and estimated the dual (right and left) GRFs using an optimization approach.

### 5.1. Ground Reaction Force

We validated our predictions of GRFs by comparing them to the experimentally measured GRF during three tasks: walking, jumping, and lifting. The predictions of the vertical component of GRF were in excellent agreement with the experimental measurements, with the rRSME ranging from 3.57% to 8.31%. Our estimations of the anterior-posterior and mediolateral components were less accurate. Previous studies also observed larger relative errors in the horizontal component of GRF [1,3,28]. These errors are attributed to the smaller magnitudes of these two components, see Figure 6. In fact, we found that their combined magnitude never exceeded 24% of the vertical component. Since the total GRF is dominated by the vertical component, this estimation error may prove acceptable in many applications. The underlying reason for the elevated sensitivity of the anterior-posterior and mediolateral components of GRF to the error compared to the vertical component lies in the fact that they are totally dependent on inertial and friction forces, while the latter is due to a combination of reactions to weight and inertial forces. Unlike weight, which is measured explicitly, inertial forces are susceptible to errors in measuring the acceleration and angular velocity and errors in estimating the segment inertial properties. Friction forces were not included in the model.

After estimating the total GRFs, feet contact with the ground was determined and used to decompose the total GRF into right and left GRFs. The vertical component of the predicted right and left GRFs showed good agreement with experimental measurements during walking with rRMSEs of 16.67% for right foot and 16.37% for left foot. However, it was not as accurate for the jumping and lifting tasks, with the rRMSE reaching as high as 48.51% for the left vertical GRF during jumping. The fundamental flaw here is the lack of a distinct single-foot stance and double-stance phases during those tasks. Both feet were continuously in contact with the ground throughout lifting and most of the jumping. Notwithstanding this limitation, the ground contact detection scheme was successful in detecting weight shifting during those tasks, interpreting weight bearing and non-weight bearing as contact and non-contact.

The force decomposition scheme was able to breakdown the total GRF into left and right GRFs. However, it was not as effective as the contact detection scheme. These errors may stem, particularly in jumping, from the impact of feet with the ground during landing. This impulsive event results in large and fast varying acceleration signals that are hard to capture accurately using the IMUs, given their sampling rate and the lowpass filter we applied at 10 Hz.

A more general limitation of the force decomposition scheme is the cost function of the underlying optimization problem. It minimizes the sum of the squared net moment in the kinematic chain of the ankle, knee, and hip joints. This corresponds to an assumption that the neuromuscular system works to minimize energy expenditure. However, the large range of flexion angles and stabilization effort required during lifting and landing from a jump undermines this assumption, since they may require synergistic muscle activation. Improving the accuracy of these predictions requires the utilization of a more elaborate cost function that better represent muscle activation in more complex tasks. In addition, the assumption of symmetry in the division of GRF between right and left GRFs has limited validity in cases involving higher accelerations such as jumping. The rRMSE of the left dominant (vertical) GRF was larger (48.51%) than its tight dominant GRF (38.8%) in jumping, while the differences during other tasks were less than 0.24%. This did not only expose a limitation in estimating the magnitude of the GRFs, but also in detecting asymmetry under this condition. The high accelerations associated with jumping may play a role in amplifying those asymmetries, as indicated by Ueberschär et al. [45], who reported 22% asymmetry in tibial vertical acceleration of 45 healthy junior-elite long-distance runners at submaximal running speeds.

Although the estimation error in our case is larger than that reported for a similar whole-body inverse dynamics analysis of the gait cycle using only measured kinematics [26], where smooth transition was assumed, the latter was only applicable for the double-stance phase of the gait cycle. Furthermore, it is only applicable to a specific motion pattern and population, where empirical data are available to allow for the derivation of a predefined load-sharing relationship.

### 5.2. Model Validation

We validated our model by comparing its estimates of the net static joint moments to those obtained from an established software, 3DSSPP. Specifically, we compared the net moment of the L5/S1, right shoulder, and left shoulder joints, which are primarily affected by the tasks under study.

Our predictions of the net joint moments were in excellent agreement with the 3DSSPP predictions for all tasks and joints, except for the L5/S1 peak moment during lifting, with the ICC = 0.9. We suggest that differences in segment mass between the models may be at the root of this disagreement.

### 5.3. Dynamic Joint Loads

The dynamic biomechanical analysis accounts for the inertial forces eliciting the additional loads imposed by motions undertaken by subjects. The dynamic loads were significantly larger than the static loads as shown in Figure 10 and Figure 11. Most notably, the dynamic to static load ratio was significantly larger for the shoulder joint than for the lower back joints. This rise was a result of faster arm movements compared to the torso as well as impact events experienced as the arms pick up, bring to a stop, and deposit a load.

### 5.4. Limitation

The number of participants (three subjects) and repetitions (three trials of each task) in the current experiment were rather small. This may be the reason for the large standard deviations of the average peak GRFs and joint moments (Figure 9 and Figure 12). Although there is a good agreement between model predictions and the ground truth reference for both male and female participants in the current study, we advise to increase the number of subjects in future studies to obtain better estimates of population-representative GRF values during those tasks.

Approximation of body segment parameters is an inherent limitation of inverse dynamics, and can lead to significant errors in estimates of joint kinetics [46]. We adopted parameters from Dumas et al. [34] definitions of the segment and their estimates of segment inertial properties. Body-scanning methods, such as those proposed by Zatsiorski [47] and de Leva [48], may be used to directly estimate individual subjects’ segment inertial properties, thereby improving the accuracy of the model estimates of the joint kinematics.

The current whole-body inverse dynamics model is also limited to the estimation of net joint moments and forces. Nonetheless, estimating individual internal forces—such as muscle contraction forces, the ligament forces and stresses, and the joint contact forces— might also be useful. Achieving this requires developing an anatomically detailed internal model for each joint [49]. These models usually apply inverse dynamics analysis of kinematics obtained from OMC systems. The model presented in this paper can also be integrated with detailed joint models for the specialized studies of the force balance at those joints.

The proposed foot contact detection method worked best during slower movements involving distinct single foot stance and double stance phases and slow foot velocity, such as walking and jogging. Its validity is limited to the detection of the weight bearing foot, in situations where a double stance dominates the time history. Furthermore, the applicability of the method should be tested for motions involving high foot velocity, such as running.

A possible improvement for the current system is to incorporate a Force-Sensing Glove for automatic detection of hand loads during material handling tasks. Adopting such a strategy will make the model more convenient to use, as it will remove the need for the user to manually input hand loads into the model. Insole pressure sensors may serve as another effective method to more accurately estimate the right and left GRFs. They may also prove useful in estimating hand loads without the need for Force-Sensing Gloves [50].

## 6. Conclusions

This study presented an inverse dynamics whole-body model that uses an IMC system only to predict joint loads. It allows for a completely portable assessment of body loads in unstructured environments unimpeded by the traditional need for motion capture cameras and force plates to conduct these analyses. It has the potential for use in risk assessment in diverse environments, including worksites. The model incorporates methods to detect foot ground contact, estimate the total GRF, and estimate the right and left GRFs during double-stance phase from body kinematics. Comparison of the model estimates for the total and right and left GRFs with those measured experimentally shows good agreement for the dominant (vertical) component of GRFs. The model was validated by comparing its predictions for the static net moments of L5/S1, right shoulder, and left shoulder joints to those of a standard static biomechanical model. Our dynamic model predictions show that static models underestimate joint loads, thereby indicating the importance of accounting for inertia in analyzing body loads.

## Figures and Tables

**Table 1:** The RMSE, rRMSE, and ICC values of the predicted total GRF components with respect to the measured total GRF components.

Truth Measure	Parameter	Walking			Jumping			Lifting		
		VE	AP	ML	VE	AP	ML	VE	AP	ML
RMSE	Total GRF (N)	37.14	53.71	39.23	126.19	98.42	111.43	84.04	60.99	88.27
		(14.04)	(25.86)	(8.67)	(55.21)	(30.54)	(37.58)	(42.99)	(21.25)	(22.13)
	Right GRF (N)	137.02	50.27	33.59	474.57	354.91	286.49	137.02	50.27	33.59
		(27.43)	(12.23)	(4.17)	(83.36)	(99.05)	(131.40)	(27.43)	(12.23)	(4.17)
	Left GRF (N)	139.76	63.16	25.77	471.98	335.02	298.60	139.76	63.16	25.77
		(30.59)	(24.62)	(6.97)	(81.96)	(91.62)	(124.78)	(30.59)	(24.62)	(6.97)
rRMSE	Total GRF	3.57	51.68	60.61	6.08	182.59	26.62	8.31	73.11	141.31
		(0.74)	(11.22)	(12.82)	(2.07)	(122.08)	(5.84)	(5.05)	(25.00)	(58.21)
	Right GRF	16.61	52.28	70.74	38.80	268.53	112.39	46.97	353.39	678.85
		(1.12)	(12.60)	(28.75)	(4.99)	(162.89)	(53.48)	(14.48)	(150.60)	(645.03)
	Left GRF	16.37	82.52	60.21	48.51	323.32	166.72	46.98	380.69	670.22
		(1.55)	(12.30)	(23.39)	(7.52)	(227.24)	(65.97)	(11.94)	(147.16)	(708.72)
ICC	Total GRF	0.98	0.58	0.91	0.95	0.79	0.62	0.92	−0.75	−0.05
	Right GRF	0.99	0.80	0.01	0.31	0.07	0.05	0.64	0.19	−0.04
	Left GRF	0.96	0.49	0.37	0.22	−0.06	0.01	0.73	0.02	0.03

RMSE = root-mean-squared error of the mean (std), rRMSE = relative root-mean-squared error of the mean (std), ICC = intraclass correlation coefficients; VE = vertical component, AP = anterior-posterior component, ME = mediolateral component.

**Table 2:** The RMSE, rRMSE, and ICC values of the IMC model-predicted static net moments at the L5/S1, right, and left shoulder joints with respect to those calculated by 3DSSPP.

Activity	RMSE (N.m)			rRMSE			ICC		
	L5/S1	Right Shoulder	Left Shoulder	L5/S1	Right Shoulder	Left Shoulder	L5/S1	Right Shoulder	Left Shoulder
Walking	2.34	0.22	0.19	11.85	5.09	6.74	0.97	0.99	0.97
	(1.86)	(0.10)	(0.09)	(6.32)	(2.61)	(4.31)			
Jumping	10.03	0.15	0.19	11.27	1.98	3.09	0.98	0.99	0.99
	(2.71)	(0.16)	(0.12)	(4.57)	(2.37)	(1.61)			
Lifting	15.20	1.01	2.27	5.44	2.88	5.89	0.90	0.97	0.93
	(9.27)	(0.49)	(0.57)	(3.31)	(1.47)	(1.97)			
